# Learning disentangled representations to harmonize connectome network measures

**DOI:** 10.1117/1.JMI.12.1.014004

**Published:** 2025-02-14

**Authors:** Nancy R. Newlin, Michael E. Kim, Praitayini Kanakaraj, The BIOCARD Study Team, Kimberly Pechman, Niranjana Shashikumar, Elizabeth Moore, Derek Archer, Timothy Hohman, Angela Jefferson, Daniel Moyer, Bennett A. Landman

**Affiliations:** a Vanderbilt University, Department of Computer Science, Nashville, Tennessee, United States; b Vanderbilt University Medical Center, Vanderbilt Memory and Alzheimer’s Center, Nashville, Tennessee, United States; c Vanderbilt University Medical Center, Vanderbilt Genetics Institute, Nashville, Tennessee, United States; d Vanderbilt University Medical Center, Department of Neurology, Nashville, Tennessee, United States; e Vanderbilt University, Department of Psychology, Nashville, Tennessee, United States; f Vanderbilt University, Department of Electrical and Computer Engineering, Nashville, Tennessee, United States

**Keywords:** connectome, brain networks, diffusion-weighted imaging, magnetic resonance imaging, harmonization

## Abstract

**Purpose:**

Connectome network metrics are commonly regarded as fundamental properties of the brain, and their alterations have been implicated in the development of Alzheimer’s disease, multiple sclerosis, and traumatic brain injury. However, these metrics are actually estimated properties through a multistage propagation from local voxel diffusion estimations, regional tractography, and region of interest mapping. These estimation processes are significantly influenced by choices specific to imaging protocols and software, producing site-wise effects.

**Approach:**

Recent advances in disentanglement techniques offer opportunities to learn representational spaces that separate factors that cause domain shifts from intrinsic biological factors. Although these techniques have been applied in unsupervised brain anomaly detection and image-level features, their application to the unique manifold structures of connectome adjacency matrices remains unexplored. Here, we explore the conditional variational autoencoder structure for generating site-invariant representations of the connectome, allowing the harmonization of brain network measures.

**Results:**

Focusing on the context of aging, we conducted a study involving 823 patients across two sites. This approach effectively segregates site-specific influences from biological features, aligns network measures across different domains (Cohen’s D<0.2 and Mann–Whitney U-test<0.05), and maintains associations with age ($2.71\times 10^{-02}\pm 2.86\times 10^{-03}$ error in years) and sex (0.92±0.02 accuracy).

**Conclusions:**

Our findings demonstrate that using latent representations significantly harmonizes network measures and provides robust metrics for multi-site brain network analysis.

## 1 Introduction

Diffusion-weighted imaging (DWI), tractography, and connectomics are advanced neuroimaging techniques that have enhanced our understanding of brain structure and connectivity.^1^^,^^2^ DWI measures the diffusion of water molecules in brain tissue, providing detailed maps of white matter fiber integrity.^3^ Tractography combines these data to estimate white matter fibers, creating comprehensive maps of brain connectivity and structures.^1^^,^^4^ Connectomics, the study of these connections, delves into the intricate network of neural interactions.^5^ Together, these methodologies are key in studying Alzheimer’s disease^6^^,^^7^ and the aging process,^8^ offering insights into the degeneration of brain networks and the consequent cognitive decline. By quantifying changes in brain connectivity with connectomics, we can better understand the progression of Alzheimer’s disease and identify potential markers for early diagnosis and therapeutic targets.^5^^,^^9^^,^^10^

**Fig. 1:**
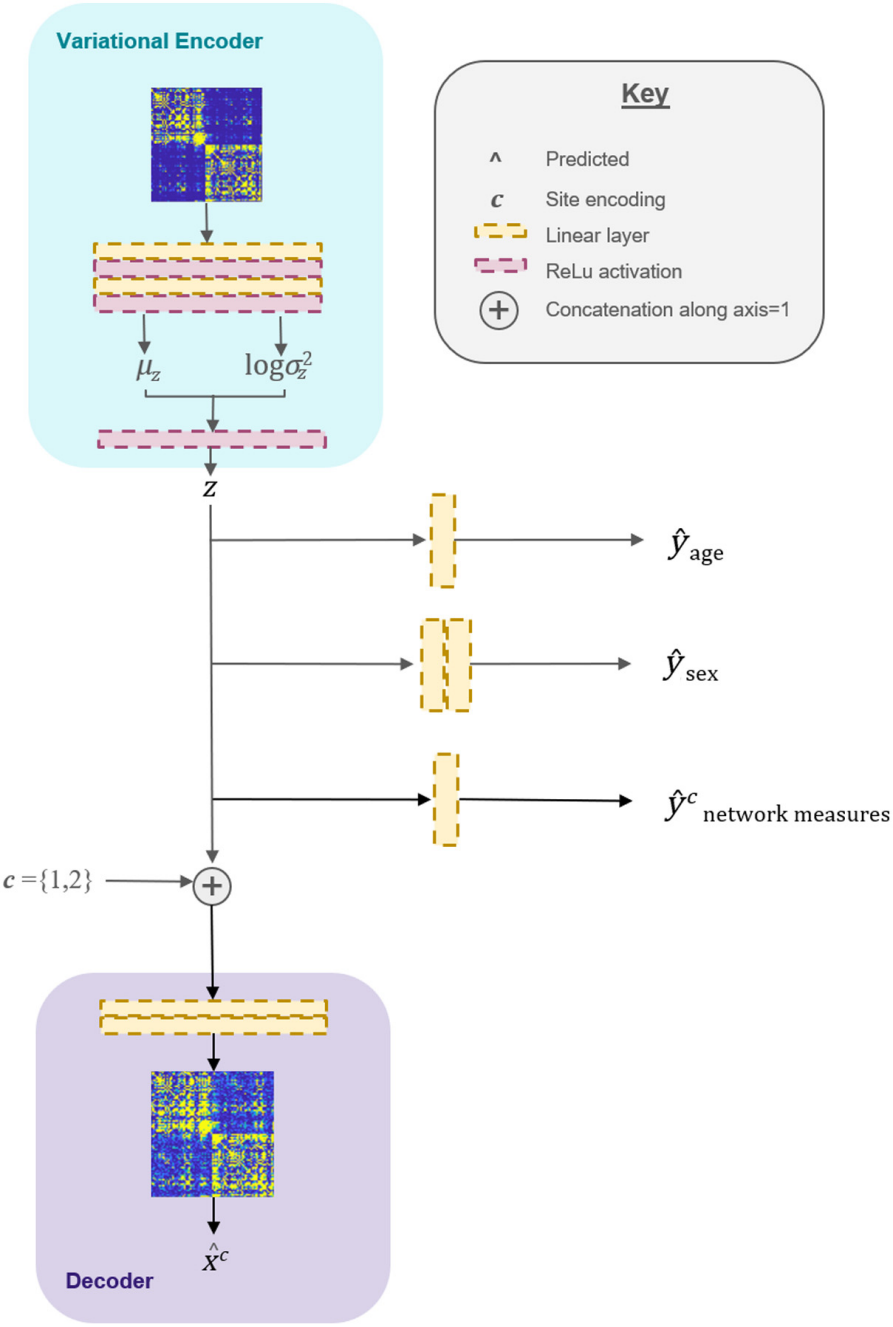
Proposed model is a VAE with a site-conditional decoder, originally proposed in Ref. 21. The encoder block comprised two linear layers and two ReLU activation functions. Following encoding, the latent space, z, is reparametrized by sampling from the learned mean ($\mu_{z}$) and variance ($\log\;\sigma_{z}^{2}$). The decoding block has two linear layers, resulting in the connectome reconstructed in the c site domain, x^c. We constrain the latent space with multiple prediction heads for patient sex (y^sex), age (y^age), and network measures (y^network measuresc).

Major hurdles in leveraging DWI to study aging and cognitive decline are small sample sizes^11^ and integrating different diffusion datasets is complex.^12^^,^^13^ There are a growing number of multi-center diffusion imaging studies that span multiple scanner manufacturers and acquisition protocols or “sites.” Alzheimer’s Disease Neuroimaging Initiative^14^ and National Alzheimer’s Coordinating Center^15^ incorporate data from multiple scanner vendors and protocols, Open Access Series of Imaging Studies^16^ includes multiple protocols, and Baltimore Longitudinal Study of Aging^17^ has data from distinct scanning hardware. Moreover, expanding DWI dataset sources to include various acquisitions, scanners, or centers introduces confounding biases due to site-specific factors. Our current network connectivity measures inherently contain such site-specific information^18^^,^^19^ (Fig. 1).

Previous efforts to address these biases have focused on non-linear harmonization in diffusion magnetic resonance imaging (MRI), primarily at the image level,^20^^–^^22^ and more broadly across MRI.^23^^,^^24^ These methods often aim to remove predictive information related to the site variable using techniques such as adversarial losses,^22^^,^^23^ variational bounds,^20^ contrastive losses,^25^ and *ad hoc* disentanglement methods.^24^ As shown in Ref. 21, optimizing these losses is the same as minimizing mutual information between a site variable and the learned representation.

The connectome is a two-dimensional graph representation that encapsulates biological and site-specific information. Each brain region translates to a connectome node, and the streamlines connecting brain regions are connectome edges.^5^ In recognizing the connectome’s potential as a lower-dimensional but rich source of connectivity information, we explore the possibility of disentangling biological and site-specific information in connectomes and extracting meaningful, site-invariant features.^26^

In this approach, we learn representations of the connectome that have disentangled site and biological features (Fig. 1). Then, we shift the representations to a common site domain to reduce confounding biases in the connectome and network measures.

## 2 Methods

**Fig. 2:**
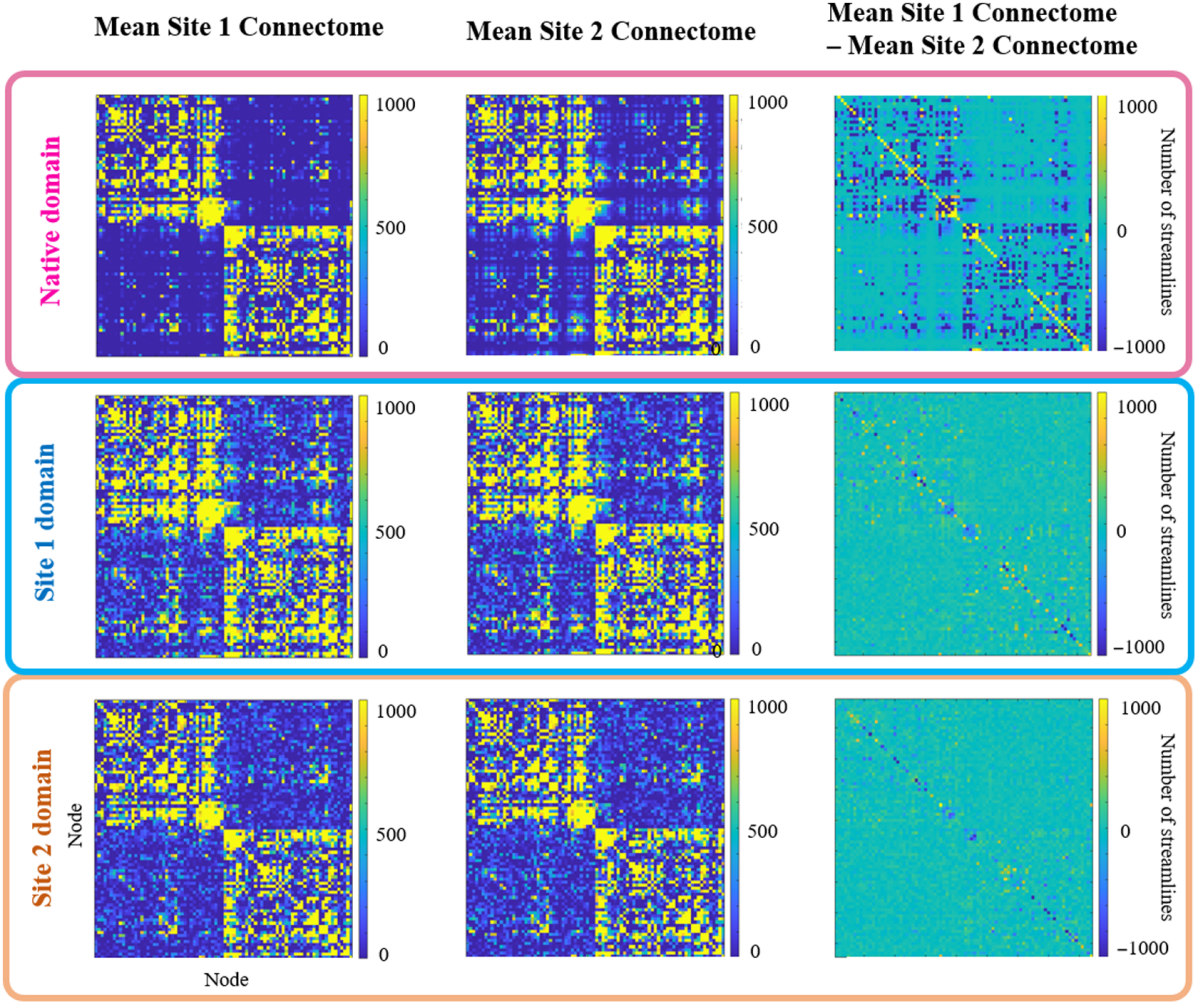
We average all connectomes from the matched testing cohort for each site. Top: Although data are matched across sites, we see systematic differences among the average connectomes, particularly in intra-hemispheric connections. We reconstruct all connectomes in a common domain to reduce inter-site differences in connectome edges. Middle: Site 1 domain. Bottom: Site 2 domain.

We propose that it is possible to disentangle information in connectomes to extract biological features with minimal or no site-specific information. Site information is a conglomeration of non-biological hardware, protocol, and study parameters that should not drive analysis.^13^ We train a neural network that produces representations that are uninformative of the site variable yet preserves relevant biological signals (Fig. 1). Then, we map this representation back to connectivity matrices conditional on a (possibly different) site variable. We show that by manipulating that site variable at test time, we choose which site domain to reconstruct (Fig. 2). Further, we can choose a common site domain for network connectivity analysis. Our method uses multi-head prediction tasks on the latent space alongside the main reconstruction task to achieve this site-minimal-bio-maximal representation.

### 2.1 Data

DWI scans from two sites were combined for joint analysis: biomarkers of cognitive decline among normal individuals: the BIOCARD cohort (BIOCARD)^27^ and Vanderbilt Memory and Aging Project (VMAP).^28^ BIOCARD patients were scanned on a 3T Philips Achieva scanner (Eindhoven, The Netherlands) at Johns Hopkins University in Baltimore, Maryland, United States. Diffusion-weighted images were acquired from a spin echo sequence (TR = 7.5 s, TE = 75 ms, resolution=0.828  mm×0.828  mm×2.2  mm, b-values=0, $700\;s/{\mathrm{mm}}^{2}$, and number of gradients = 33). VMAP patients were scanned on a Philips 3T Achieva scanner (Best, The Netherlands) at Vanderbilt University in Nashville, Tennessee, United States. Diffusion-weighted images were acquired from a spin echo sequence (TR = 8.9 ms, TE = 4.6 ms, resolution=2  mm×2  mm×2  mm, b-values=0, $1000\;s/{\mathrm{mm}}^{2}$, and number of gradients = 32).

Data used in this study are split into training and testing cohorts. The training cohort comprised 347 scans from BIOCARD (site 1) and 324 scans from VMAP (site 2), all free of cognitive impairment. Training data from VMAP have ages 74.1±7.5 and 114 women. BIOCARD training data are ages 73.1±5.7 with 205 women. The testing cohort is 77 matched participants (one scan from each participant), free of cognitive impairment, ages 72.9±7.6, and 57% percent women.

To isolate site-wise differences between these two datasets without traveling subjects,^29^^,^^30^ we curate a subset of patients that have the same demographics. The matching process finds a 1:1 cross-site mapping of each patient based on demographics. For example, a 59-year-old female from the VMAP cohort is matched with a 59.5-year-old female from the BIOCARD cohort. Matched patients are the same sex and ±1 year in age. The matching was done using the pyPheWAS maximal group matching tool (version 4.1.1).^31^

### 2.2 Diffusion Processing

The proposed model learns from connectome representations of diffusion tractography and is derived from DWI outlined in Sec. 2.1. DWI from all participants were first preprocessed to remove eddy current, motion, and echo-planar imaging distortions prior to any model fitting.^32^ We used MRTrix^33^ to perform tractography over fiber orientation distributions with anatomically constrained tractography framework (seeded on gray matter–white matter interface, allowed backtracking, terminating with five-tissue-type mask, and generated 10 million streamlines^34^). Afterward, we map the tractogram to a connectome representation using the Desikan–Killany atlas^35^ with 84 cortical parcellations from Freesurfer.^36^ We use the Brain Connectivity toolbox (version-2019-03-03) to compute 12 brain network measures^5^ and filter connections less than 0.00001% of the total number of streamlines.^34^ Network measures computed with this toolbox are the site-biased ground truth used in model training.

We consider modularity, average node betweenness centrality, assortativity, average node participation coefficient, average node clustering, average node strength, average local efficiency, global efficiency, density, rich club coefficient, characteristic path length, and number of edges in the characteristic path length (“edge count”).^5^ Modularity is the quality of division of the network into modules.^5^ Betweenness centrality is the sum of the ratio between the shortest paths that contain the node and the total number of the shortest paths.^5^ Assortativity is the correlation coefficient among the degrees of all nodes on two opposite ends of a connection and reflects network resilience.^5^ Participation coefficient characterizes the diversity of intermodular connections of nodes.^5^ Clustering coefficient is the possibility that any two neighbors of a node are also connected.^5^ Here, node strength is the number of streamlines connecting a node.^5^ Global efficiency is the ability for information to move around the whole network, and local efficiency is the ability of information to move around nodal subsets.^5^ Density is the number of present connections to the total number of connections possible.^5^ Rich club networks have subgroups of central nodes that tend to interact with one another.^37^ The characteristic path length is the average shortest path, in millimeters, to traverse the network.^5^

### 2.3 Model Architecture

We implemented a variational autoencoder (VAE) with site-conditional restrictions on the latent space, z. The proposed model architecture is outlined in Fig. 1. First, the encoder processes the input data using two layers of linear layers and two layers of non-linear activation (ReLU). Following encoding, the latent space is reparametrized by sampling from the learned distribution. Next, the decoder takes this compact representation and reconstructs it back into the original data format while accounting for site-specific characteristics. In addition to recreating the original connectome, the model is trained to predict other useful information, such as the patient’s sex, age, and brain connectivity features. These predictions help ensure that the latent space is organized in a way that captures meaningful patterns and relationships in the data. During test time, we set the decoding conditions to project network measures and the reconstructed connectome to a site domain of the user’s choice.

We chose this architecture to learn site-minimal, bio-maximal representations of the connectome. We accomplish this goal by maximizing mutual information (I(x,y)) of z and biological attributes (let a be patient age and s be patient sex) and z with network connectivity properties (B). Simultaneously, we minimize mutual information of z with site (let c be the site encoding). We translate these relationships to loss functions as follows: to maximize mutual information, we minimize loss [mean squared error (MSE) or binary cross entropy]. To minimize mutual information between z and site, we minimize MSE loss of the conditional reconstruction [the first term in Eq. (1)] and maximize KL divergence [the second term in Eq. (1)].

I(z,c)≤log(p(x|z,c))−KL[q(z|x)|q(z) (1)

The second term in this bound is difficult to compute directly but, as shown in Moyer et al.,^20^ can be approximated using the standard normal Gaussian in place of induced marginal q(z). This fits nicely with existing VAE literature,^38^ is computationally tractable, and, as we show, performs well empirically for removing site information. We use Eq. (1) to replace I(z,c) in Eq. (2).

Therefore, the overall loss function, $\ell_{\text{total}}$, is the sum of five sub-component losses: connectome reconstruction ($\ell_{\mathrm{recon}}$), mean squared site-conditional prediction error for brain network measures for BIOCARD ($\ell_{B}(c)$, c=1) and VMAP ($\ell_{B}(c)$, c=2), mean squared age prediction error ($\ell_{\text{age}}$), and binary cross entropy loss of sex predictions ($\ell_{\text{sex}}$). The final sub-component loss is KL divergence, which is a key component in our learning as it limits site information in z.

Toward that end, our component loss function can be rewritten as mutual information terms (up to constant entropic terms):

$$I(z,c)-I(z,B)-I(z,a)-I(z,s)\;=\ell_{\mathrm{recon}}(c)+\ell_{\mathrm{kl}}+\ell_{\text{age}}+\ell_{\text{sex}}+\ell_{B}(c)\;=\ell_{\text{total}}$$ (2)

### 2.4 Co-Learning and Individual Learning Schemes

We explore two learning schemes for optimizing the latent space. In the first scheme, the model learns to predict one brain network measure from the site-invariant latent space (“individual learning”). We then train 12 separate models, one for each brain network measure. In the second scheme, the model learns to predict all 12 brain network measures at the same time from the site-invariant latent space (“co-learning”).

### 2.5 Bootstrapping

To assess training stability, we bootstrapped training data. At each bootstrap iteration, 80% of training data are randomly sampled without replacement. The same testing data are used to evaluate each iteration.

### 2.6 Evaluating Harmonization Performance

We measure successful harmonization by evaluating differences in the connectomes and network measures with respect to their site variable. Although we do not use traveling participants in this study, we do match patients across sites based on their age, sex, and cognitive status. We expect network measures computed for this matched dataset to have similar distributions. We measure the disparity between sites with Cohen’s D and the Mann–Whitney U-test of medians (with p-value<0.05 as significant). Cohen’s D is a standardized effect size that reports the difference between two means compared with the overall data variability.^39^ Successful harmonization would reduce large (Cohen’s D>0.5) differences to small (Cohen’s D<0.2) and remove significant differences in the medians among sites.^39^

The second benchmark of harmonization performance is preserving biological variation. Previous studies^40^^–^^42^ demonstrated that connectomes contain relevant signals related to patient age and sex. During model training, the latent space is shaped by five tasks. Two of which, sex and age prediction, are included to encourage the model to retain relevant biological covariates.

## 3 Results

**Fig. 3:**
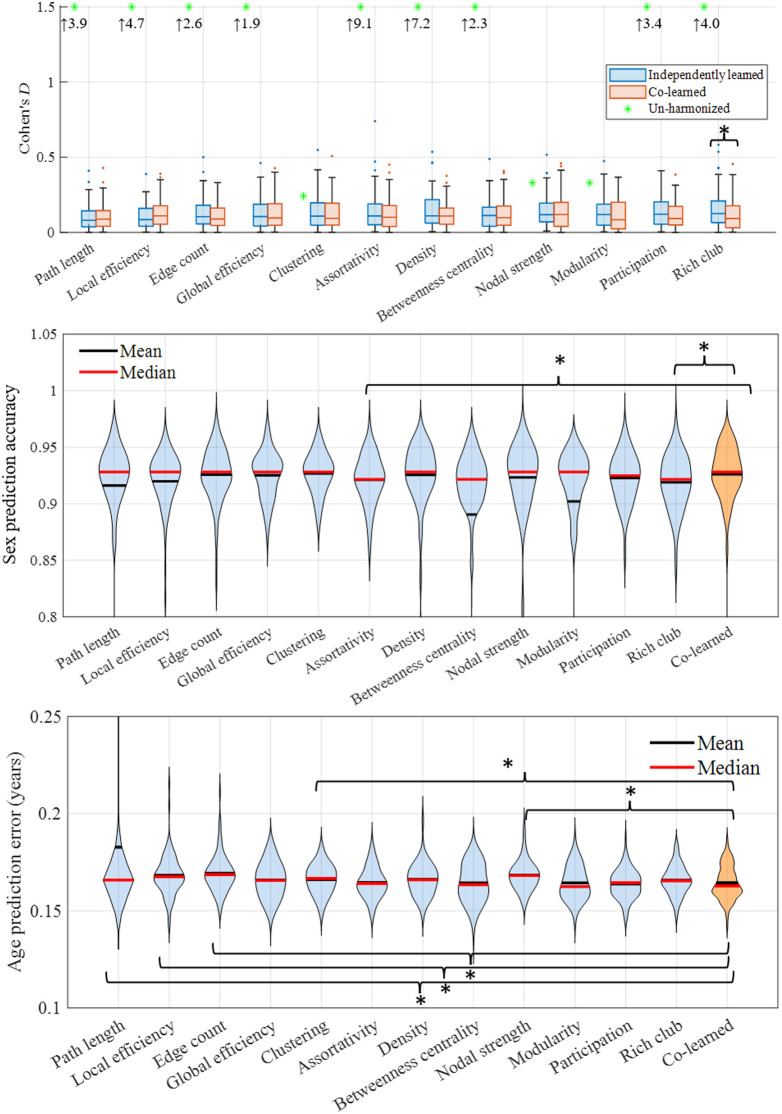
We evaluate the harmonization efficacy of the learning site domain of each network measure separately (blue) and co-learned together (orange) across 100 bootstrapped experiments. Both schemes reduce inter-site differences to small and medium effect sizes by projecting data to a common site (site 1, left; site 2, right); however, co-learning improves performance in measures marked with significant differences in medians (*p<0.05). In addition, the co-learning structure has comparable or improved sex prediction accuracy (middle) and age prediction error (bottom).

**Fig. 4:**
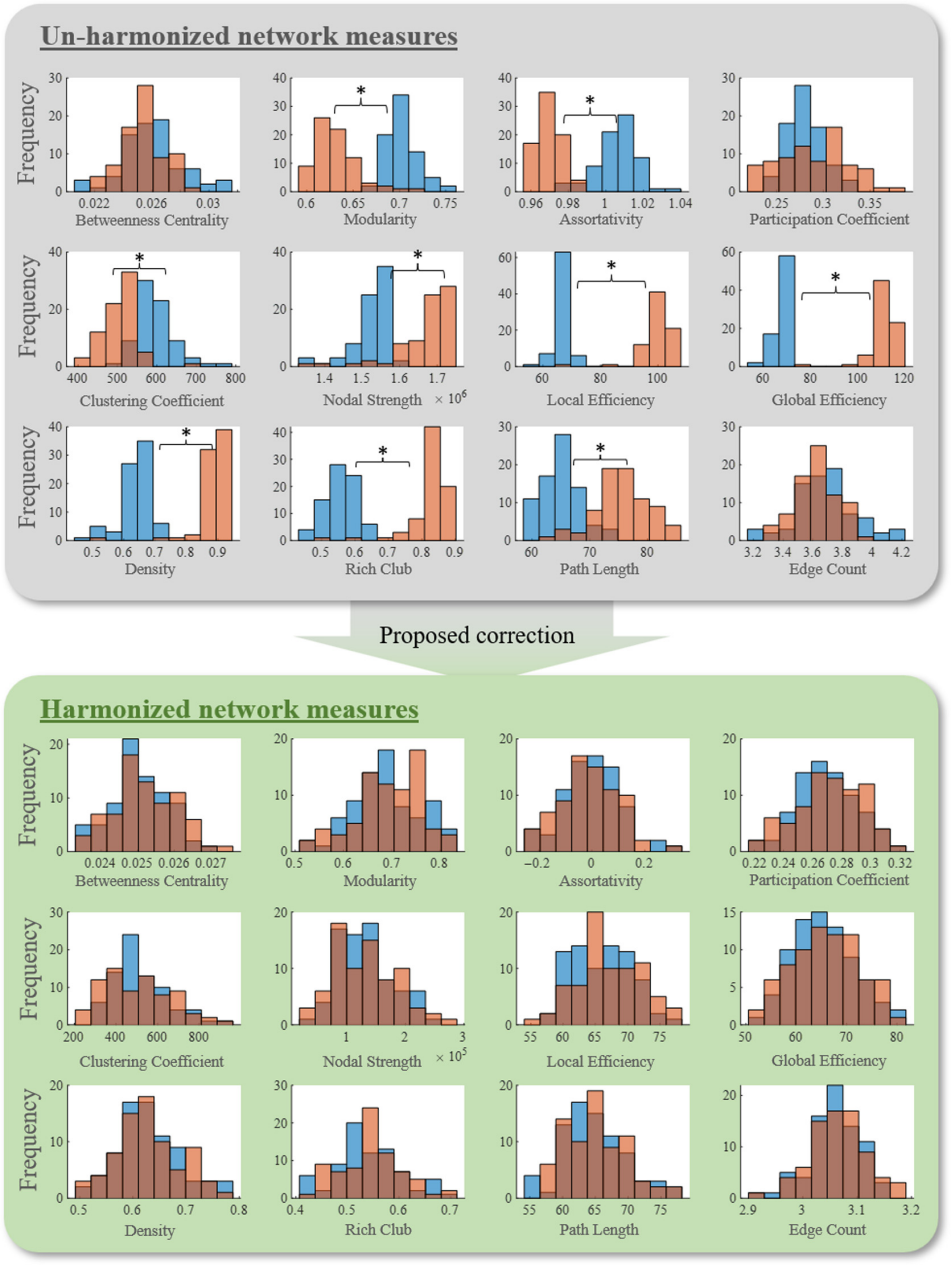
Here, we project network measures for the demographically matched testing set to site 1 using the proposed correction method. We show histograms of site 1 values originally from the BIOCARD cohort (blue) and site 2 values originally from the VMAP cohort (orange) before and after correction. Significant inter-site differences (*p-value<0.05) in the median are ameliorated in the domain-shifted network measures.

Although data are matched across sites, we observe systematic differences among the average uncorrected connectomes, particularly in intra-hemispheric connections (Fig. 2). The model successfully projects connectomes and brain network measures to a common site domain. Inter-site differences in all network measures are reduced to small and medium effect size differences (Fig. 3). In addition, significant site-wise differences in the median in modularity, assortativity, average node clustering coefficient, average node strength, average node local efficiency, global efficiency, density, rich club, and characteristic path length are no longer significant (Fig. 4).

Individually and co-learned models retain biological information while reducing inter-site variation (Fig. 3). Under the co-learning scheme, patient sex is predicted with 0.92±0.02 accuracy, and patient age is predicted with $2.71\times 10^{-02}\pm 2.86\times 10^{-03}$ error in years.

## 4 Discussion

There is a growing need for DWI-derived features that can be used reliably in multi-site studies. Harmonization methods play a critical role in pooling data from multiple sites with varying sample sizes and age ranges, enabling the study of complex pathologies that would otherwise suffer from limited statistical power and generalizability.^13^ This need is particularly acute in Alzheimer’s disease research, where cohort sizes are typically small.^11^ These cohorts require harmonization techniques that are robust to variations in sample size and overlapping age distributions.^13^ To address this, we propose a method that extracts site-invariant features and harmonized network measures, serving as quantitative tools for multi-site analyses of neurodegeneration and aging.

A key advantage of our method is its independence from traveling subjects or perfect demographic matching, which are often impractical requirements. Although we used demographic matching to validate harmonization performance, the model itself does not require matched data during training or testing. This flexibility makes our approach more scalable and applicable to diverse datasets. Currently, the model has been calibrated using connectomes from patients without cognitive impairment across only two sites. Future work will aim to expand its applicability to patients with cognitive impairment and extend its utility to datasets spanning multiple sites.

One of the most widely used harmonization methods is ComBat, which estimates and reduces site-specific effects on the sample mean and variance.^43^ Although efficient and lightweight, ComBat is only reliable for datasets with more than 162 scans and minimal mean age differences among sites.^44^ These limitations restrict its utility in studies with smaller cohorts or significant demographic variations.

An alternative to ComBat harmonization is calibrating scanner differences using rotationally invariant features of DWI rather than downstream features.^45^^,^^46^ This method involves computing the average rotationally invariant signal for each site and generating a multiplicative template to normalize the signals across sites.^45^ Although this approach ensures consistency in signal intensities, it requires all images to be co-registered to a common space. This step is computationally intensive and can result in a significant loss of information. Furthermore, it necessitates demographic matching across sites, which poses additional challenges in multi-site studies.^45^

On the whole, our proposed method offers a promising solution for harmonizing multi-site DWI data by extracting site-invariant features and harmonized network measures. Unlike existing methods such as ComBat or rotationally invariant signal calibration, our approach balances computational efficiency with robustness to demographic mismatches. By enabling the integration of diverse datasets, our method holds the potential for advancing the study of neurodegeneration and aging in a multi-site context.

## 5 Conclusion

We assert the efficacy of a conditional variational autoencoder for minimizing mutual information between the latent space and site variable. We explored two schemes for further optimizing the latent space by maximizing information related to network connectivity and biological covariates. Co-learning has improved harmonization efficacy and biological preservation over the individually learned scheme. With the proposed model, we extract site-invariant connectome features that are predictive of brain network structure, patient sex, and patient age.

## Data Availability

Code is publicly available at https://github.com/nancynewlin-masi/LearningSiteInvariantConnectomeFeatures.
